# Fluorescence lifetime imaging for intraoperative cancer delineation in transoral robotic surgery

**DOI:** 10.1002/tbio.201900017

**Published:** 2019-10-29

**Authors:** Brent W. Weyers, Mark Marsden, Tianchen Sun, Julien Bec, Arnaud F. Bewley, Regina F. Gandour-Edwards, Michael G. Moore, D. Gregory Farwell, Laura Marcu

**Affiliations:** 1 Department of Biomedical Engineering, University of California, Davis, California; 2 Department of Computer Science, University of California, Davis, California; 3 Department of Otolaryngology, University of California, Davis, California; 4 Department of Pathology and Laboratory, Medicine, University of California, Davis, California

**Keywords:** autofluorescence, coregistration techniques, fluorescence lifetime imaging, head and neck cancer, intraoperative surgical guidance, robotic surgery, surgical visualization

## Abstract

This study evaluates the potential for fluorescence lifetime imaging (FLIm) to enhance intraoperative decisionmaking during robotic-assisted surgery of oropharyngeal cancer. Using a custom built FLIm instrument integrated with the da Vinci robotic surgical platform, we first demonstrate that cancer in epithelial tissue diagnosed by histopathology can be differentiated from surrounding healthy epithelial tissue imaged *in vivo* prior to cancer resection and *ex vivo* on the excised specimen. Second, we study the fluorescence properties of tissue imaged *in vivo* at surgical resection margins (tumor bed). Fluorescence lifetimes and spectral intensity ratios were calculated for three spectral channels, producing a set of six FLIm parameters. Current results from 10 patients undergoing TORS procedures demonstrate that healthy epithelium can be resolved from cancer (*P* < .001) for at least one FLIm parameter. We also showed that a multiparameter linear discriminant analysis approach provides superior discrimination to individual FLIm parameters for tissue imaged both *in vivo* and *ex vivo*. Overall, this study highlights the potential for FLIm to be developed into a diagnostic tool for clinical cancer applications of the oropharynx. This technique could help to circumvent the issues posed by the lack of tactile feedback associated with robotic surgical platforms to better enable cancer delineation.

## Graphical Abstract

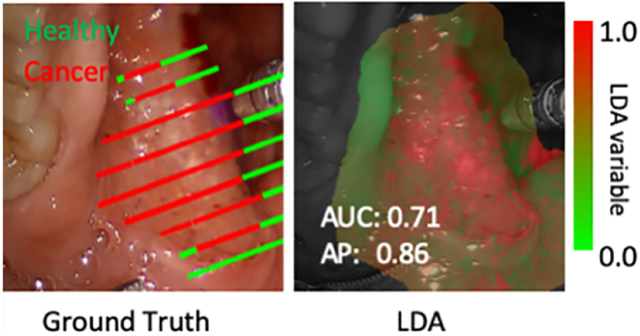

## 1 | INTRODUCTION

In the United States, oral and oropharyngeal cancer together represent 3.0% of all new cancer cases, are associated with a 65.3% survivability after 5 years from initial onset, and are expected to afflict 53 000 individuals in 2019 [1]. Based on global trends, oropharyngeal cancer comprises approximately half of these cases [2]. Over the last two decades, robotic-assisted surgery has become widely utilized for the surgical resection of oropharyngeal cancers. Transoral robotic surgery (TORS) confers many advantages compared to conventional endoscopy procedures; this includes deeper access to anatomical sites which enables precise operation in tight spaces without a large open incision, improved patient functional outcomes, and improved dissection ability of lesions and neoplastic growths [3].

Adequate intraoperative delineation of cancer is the key factor for long-term survival of patients diagnosed with oral and oropharyngeal cancer [4]. This requires rapid evaluation of the extent of molecular changes (neoplastic area) of the epithelial surface (mucosa). The traditional gold standard for intraoperative oral and oropharyngeal cancer delineation is white-light visualization, tactile feedback, and pathologic consultation through a combination of techniques including frozen section histopathology [5]. During conventional surgical resection procedures, surgeons typically leverage all three methods, as well as other screening tests, to provide informed intraoperative diagnosis [5].

The current approach for TORS presents two fundamental limitations. First, the TORS platform eliminates the surgeon’s ability to sense tissue and bone resistances, which creates a loss of haptic feedback [3, 6, 7]; this limitation has been cited to make TORS procedures more challenging [6, 7]. Second, the reliance of frozen section analysis during TORS procedures introduces long procedural waiting times, sampling error, the inability to provide continuous assessments of pathology margins, invasiveness associated with biopsies, and the potential for interpretative errors [8].

Alternative techniques for real-time intraoperative cancer delineation in oral anatomy have been evaluated and reported to address such intraoperative challenges [9–12]. This includes fluorescence spectroscopy and imaging techniques based on exogenous (using both non-targeted and targeted probes) [13, 14] and endogenous [15–17] fluorescence.

Recently, there has been in increased interest in the development of autofluorescence techniques for intraoperative use [12, 18]. The strong emission of endogenous fluorophores (e.g. nicotinamide adenine dinucleotide [NADH] and flavin adenine dinucleotide (FAD) in the epithelial cell layers, collagen crosslinks in the stroma upon UV-vis excitation) were previously exploited and shown to improve the ability to distinguish normal from premalignant or malignant oral tissue in humans [18]. For example, autofluorescence devices for early detection of oral neoplasia (including the FDA approved VELscope, LED Dental Inc.) have been reported previously [9, 10, 12, 13, 19]. Although, these devices rely on the shallow penetration (<400 μm) of UV-Vis excitation light, it has been shown that they can detect changes in the epithelium with high sensitivity [18]. However, autofluorescence techniques based on steady-state fluorescence intensity analysis are confounded by factors such as irregular tissue surfaces (excitation/collection geometry), non-uniform tissue illumination, and variable presence of endogenous absorbers such as blood in the operative field [17]. Accordingly, lifetime-based autofluorescence analysis has been identified as an attractive alternative due to its insensitivity to the confounding factors of intensity-based analysis.

Tissue autofluorescence time-resolved measurements have recently demonstrated potential to delineate cancer intraoperatively from surrounding normal tissues in patients [20–23], including oral cancer [20, 24, 25]. Time-resolved autofluorescence techniques can address limitations of the steady-state based methods by resolving the dynamics of the fluorescence decay (lifetime) [23]. By taking advantage of alterations in tissue structural and metabolic characteristics associated to neoplastic processes [23, 26], lifetime-based methods have the potential to provide information about tissue molecular composition, including enzyme cofactors involved in cellular metabolism (eg, NAD(P)H and FAD), matrix proteins (collagen and elastin), and inflammatory activity.

In a recent study [20], we demonstrated the integration of a fluorescence lifetime imaging (FLIm) system into the da Vinci Surgical System, and highlighted its functionality during conventional TORS procedures. Herein, this work showed that FLIm-derived parameters can discriminate between different tissue types at the epithelial surface during oropharyngeal cancer procedures, including carcinoma, carcinoma over lymphoid tissue, and normal tissue [20]. However, we noted that optical/fluorescence parameters can be affected by a variety of experimental situations. This includes diverse tissue types (e.g. tumor of distinct phenotypes or heterogeneity) and conditions (thickness of the epithelial layer, presence of lymphoid tissue, approach to tumor removal, cautery, and hemostasis).

The goals of this study were to conduct FLIm measurements during conventional TORS procedures, to evaluate the effects of experimental procedures on FLIm data, and to determine whether a combination of FLIm-derived parameters can be always found and used as means of intrapatient diagnostic contrast irrespective of experimental situations. For this, we evaluated the ability of a multiparameter discrimination approach that uses a set of spectral and time-domain fluorescence parameters to resolve healthy epithelium from cancer tissue imaged both *in vivo* prior to resection and *ex vivo* in surgically-excised specimens. Multiparameter and single parameter approaches were compared. In addition, we sought to investigate the FLIm characteristics of the tumor bed imaged post-resection and to perform quantitative comparisons with other imaged tissue types (*in vivo* healthy epithelium and *in vivo* cancer).

## 2 | MATERIALS AND METHODS

### 2.1 | Instrumentation

#### 2.1.1 | FLIm device

**FIGURE 1:**
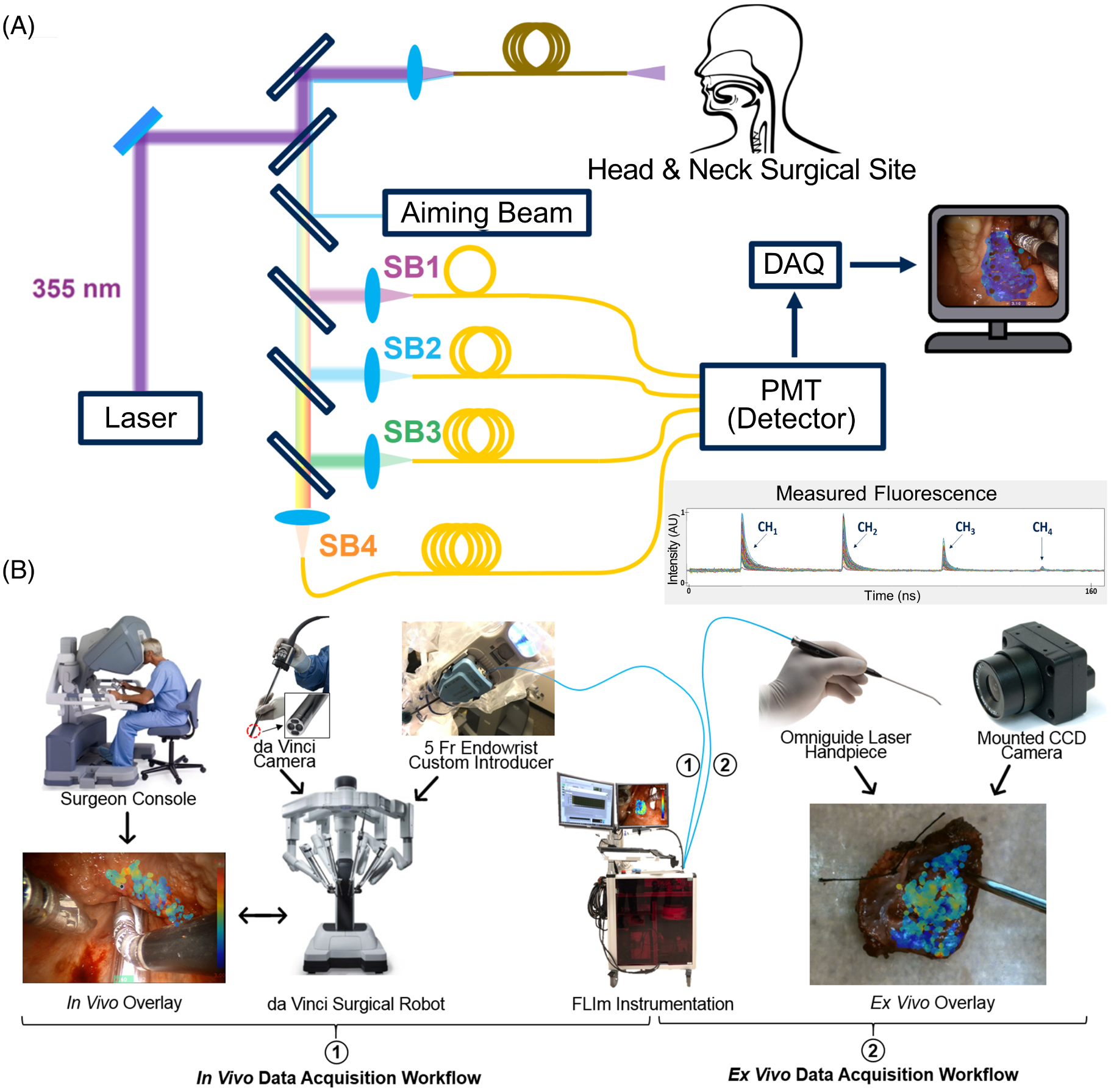
Overview of the fluorescence lifetime imaging (FLIm) instrumentation and workflow in the operating room. (A) Schematic of the custom-built FLIm system, featuring the excitation beam to generate autofluorescence, the aiming beam to spatially coregister data, and the four spectral channels to resolve fluorescence lifetime and spectral intensity. Also illustrated is an example of the measured fluorescence waveforms output from the four time-delayed spectral channels; the method for the detailed calculation of fluorescence lifetime and spectral intensities for each spectral channel is described by Liu et al. [27]. (B) Integration of the FLIm system with the da Vinci robotic system in the OR workflow: (1) represents the *in vivo* workflow for both pre-resection and post-resection (cavity) analysis where the da Vinci surgical system (including the integrated camera) was leveraged to collect measurements, and (2) represents the *ex vivo* workflow used for resected specimen pathology assessments where an Omniguide Laser Handpiece was used to perform a hand-held scan visualized by a mounted camera. The surgeon console and da Vinci system images are adapted with permission from Intuitive Surgical Inc

A custom-built fiber-based point-scanning FLIm system was coupled to the da Vinci Si Surgical System via a 5Fr EndoWrist Introducer (schematic depicted in Figure 1A) as previously described [20]. In brief, tissue autofluorescence was excited with a 355 nm (<600 ps FWHM) pulsed laser (micro Q-switched laser, 120 Hz repetition rate, Teem Photonics, France) delivered through a 365 μm core multimode optical fiber inserted in the EndoWrist Introducer. The same fiber optic was used to collect the autofluorescence emanating from the tissue regions evaluated. The fiber’s proximal collection end was coupled to a wavelength selection module (WSM) which features a set of four dichroic mirrors and bandpass filters (i.e. CH1: 390 ± 20 nm; CH2: 470 ± 14 nm; CH3: 542 ± 25 nm; and CH4: 629 ± 26.5 nm) used to spectrally resolve the autofluorescence signal. These spectral bands were tailored to capitalize on the autofluorescence emission maxima of endogenous fluorophores previously reported as the main contributors to head and neck cancer autofluorescence emission, specifically collagen, NAD(P)H, FAD, and porphyrins [23]. The optical signal from each spectral band was time-multiplexed into a single microchannel plate photo-multiplier tube (MCPPMT, R3809U-50, 45 ps FWHM, Hamamatsu, Japan), amplified (AM-1607–3000, Miteq Inc., USA), and time-resolved by a high sampling frequency digitizer (12.5 GS/s, 3GHz, 8-bit, 512 Mbytes, PXIe-5185, National Instruments, Austin, TX, USA) at 80 ps time intervals.

The RF amplifier was AC coupled with a low cutoff frequency of 10 kHz which filters out any signal contribution from the continuous-wave aiming beam and operating room lights [28]. The principle behind this technique was described in detail in our earlier work [28, 29]. *In vivo* and *ex vivo* data were collected using configurations 1 and 2 respectively, as highlighted in Figure 1B.

#### 2.1.2 | FLIm point-measurement localization

**FIGURE 2:**
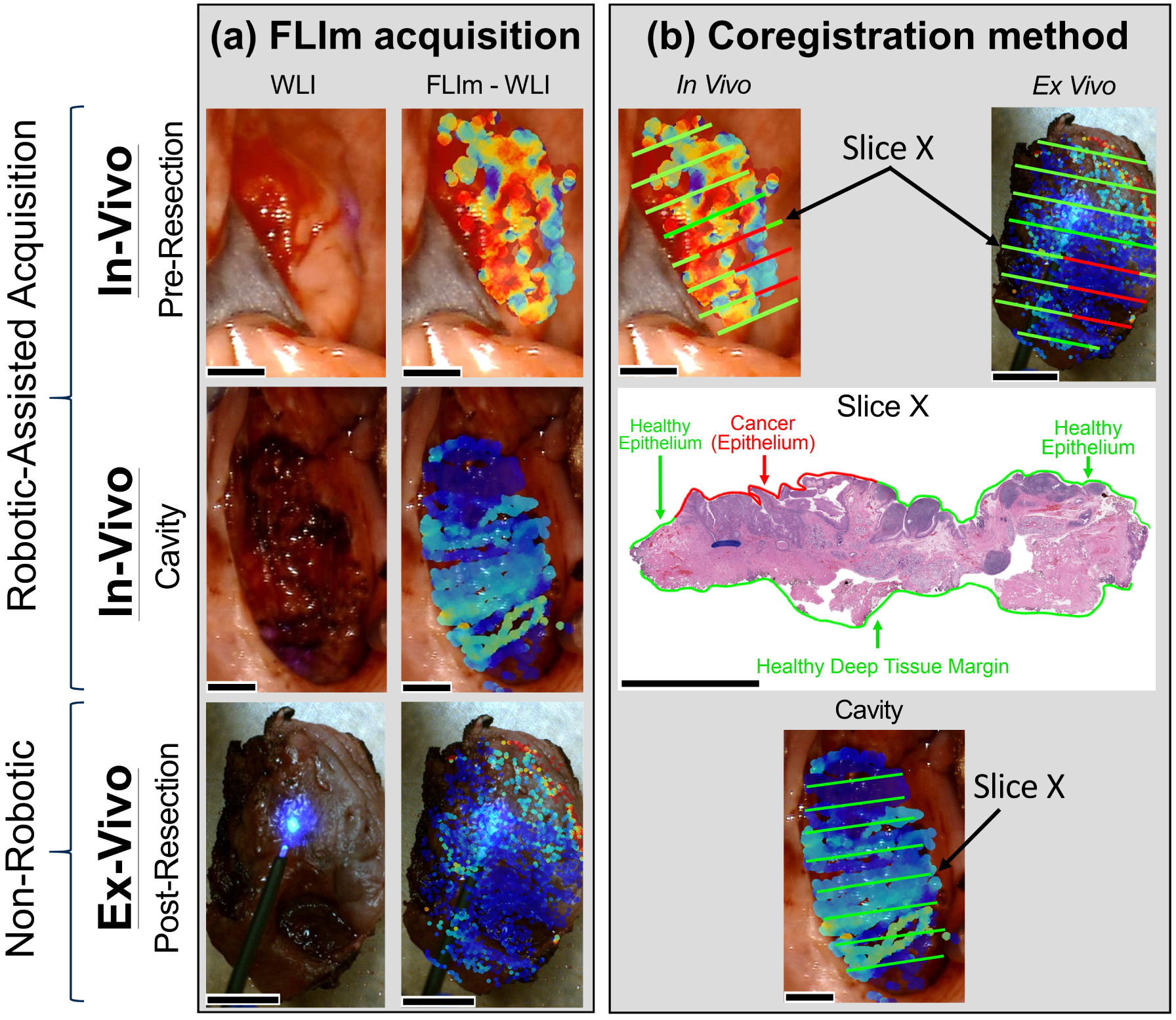
Method for fluorescence lifetime imaging (FLIm) data acquisition and coregistration with tissue histopathology. (A) First, the *in vivo* pre-resection region of interest was identified by the surgeon (DGF, AFB, and MGM) as demonstrated in the white light image (WLI). A FLIm scan was performed at the anticipated cancer location to generate a FLIm-augmented WLI. Peripheral healthy tissue surrounding the cancer region was also included in the scan, where peripheral tissue was defined as a 10 pixel distance from the surgical margins (images are 1280 × 720 pixels for all scan types). Ten pixels correspond to approximately 0.75 mm. Second, following the cancer resection (facilitated by the da Vinci robot surgical platform) an *in vivo* post-resection scan was performed at the resection cavity. Surrounding peripheral tissue which was unaltered by the procedure was also included in the scan. Third, an *ex vivo* post-resection scan was performed on the surgically excised specimen. (B) In order to coregister FLIm data to histology, the surgically excised *ex vivo* specimen was first sliced in a grossing room under the surgeon’s direction and careful notes were acquired regarding the sectioning process. The sections (such as “Slice X” denoted in the figure tile) were then stained with hematoxylin and eosin and P16 antibodies, the pathologist (RGE) annotated the corresponding histology, and the results were extrapolated onto the respective slice *ex vivo*. The spatially oriented *ex vivo* specimen, along with clinical notes from the surgeon and pathologist, were used to perform subsequent coregistration *in vivo* at the pre-resection location using pathology annotations from the tissue surface. Post-resection (cavity) coregistration was performed using pathology annotations from the deep tissue margin (as indicated on the “Slice X” histology). All scale bars represent 0.5 cm

To determine the spatial location of each FLIm point measurement, we employed a previously reported method [28]. Specifically, a 455 nm continuous-wave aiming beam (TECBL50G-440-USB, World Star Tech, Canada) was injected into the WSM optical path and delivered to tissue through the same fiber optic used to induce and collect tissue autofluorescence [28]. Then, the position of the aiming beam was localized within a two-dimensional (2D) white light image of the tissue specimen captured by the camera integrated into the da Vinci system. This localization was performed by transforming the image into the HSV color space, thresholding the hue and saturation channels, and performing a series of morphological operations to isolate the center of the beam [28]. By performing aiming beam segmentation in parallel with the deconvolution of autofluorescence decay signals, FLIm parameter visualizations can be generated. This approach is depicted in Figure 2. A mounted camera was used for aiming beam localization for *ex vivo* measurements of excised tissue specimens.

### 2.2 | Human patients and data collection

Under Institutional Review Board (IRB) approval, 10 human patients undergoing upper aerodigestive onco-logic surgery (using the da Vinci Surgical System at the University of California Davis Medical Center) were recruited from the Otolaryngology Head & Neck Surgery clinic after determination of their eligibility for this research. The patients enrolled in this study, along with their corresponding surgical locations, etiologies, and residual cancer status are enumerated in Table 1. Research was conducted on patients only after their informed consent was obtained.

Prior to robotic surgery, the EndoWrist instrument containing the fiber optic from the FLIm instrument was installed in the da Vinci surgical robot and was placed inside the oral cavity of the patient under anesthesia. Surgeons (DGF, AFB, MGM) identified the tissue areas of interest based on preoperative planning. FLIm measurements were then acquired by scanning the EndoWrist instrument over that region. FLIm data was collected during three distinct stages of surgical resection (Figure 2A): (a) *in vivo* prior to tumor excision from anticipated cancer locations and the surrounding healthy peripheral tissue (defined as a 10 pixel distance from the surgical excision margins which corresponds to approximately 0.75 mm), (b) *in vivo* after surgical tumor excision in the resection bed, and (c) for the excised *ex vivo* specimen. The average time spent to map out an entire tissue specimen (and neighboring periphery if *in vivo*) is approximately 0.75 to 1.5 minutes depending on the tissue area. Immediately following an *ex vivo* scan, the resected specimen was sent for sectioning and histopathology staining. Hematoxylin and eosin and P16 immunostaining was used as the primary agent for pathology detection. The fixed sections were placed on slides, scanned for virtual retrieval using an Aperio digital pathology slide scanner (Leica Biosystems), and annotated by a pathologist (RGE) as illustrated in Figure 2B. Careful notes were taken to designate the precise location where histological sections were taken from the excised sample to coregister the pathologist’s annotations to the *ex vivo* sample. The spatially orientated *ex vivo* specimen, along with clinical notes from the surgeon and pathologist, were used to perform coregistration of the *in vivo* images at the pre-resection and post-resection (tumor bed) locations as illustrated in Figure 2B. The operating surgeon was consulted during each procedure to assist with orienting the excised specimen on *in vivo* anatomy. The pathologist was not presented with any FLIm results in order to prevent any potential bias in the pathology interpretation.

### 2.3 | Data analysis and visualization

#### 2.3.1 | Deconvolution and FLIm parameter extraction

Following the acquisition of the raw fluorescence decay signal, background subtraction was applied to each spectral channel. The extraction of FLIm parameters including spectral intensity ratio and average fluorescence lifetime values was performed for each channel using a constrained least-squares (CLS) deconvolution via Laguerre expansion as described previously [27]. This method allows for fast and accurate analysis of fluorescence decay dynamics and for real-time computation of fluorescence lifetime values and other relevant decay parameters. Intensity ratios were calculated for a given spectral channel by dividing the integral of that channel’s fluorescence decay curve by the sum of decay curve integrals for all channels.

#### 2.3.2 | FLIm parameter visualization

Once a set of FLIm measurements and (*x*,*y*) locations have been acquired for a given scan, each of the calculated signal parameters can be visualized through the generation of a parameter heat map. Each heat map can be overlaid separately onto the captured white light image to highlight how each parameter varies across the scanned tissue. A pixel-wise interpolation method was used to combine the inverse distance weighted interpolation of Shepard’s Method [30] with signal-to-noise ratio (SNR) weighting. Measurements with a close spatial proximity to a given pixel of interest and a high SNR value have the most influence on the final output. The applied visualization method was implemented in the Python programming language and employs the OpenCV image processing library [31].

#### 2.3.3 | Data annotation, coregistration, and preprocessing

Histopathology annotations were performed for each case using a custom software tool developed in MATLAB. Here, a set of pixel-wise annotations were generated for a given scan and represented as a gray scale image where each pixel value (0–255) corresponds to a specific tissue condition. Four total pixel values were used in this study for coregistration analysis to designate: (a) no coregistration annotation, (b) cancer, (c) healthy epithelium, and (d) electrocauterized submucosa (tumor bed). For this study focused on intrapatient contrast, only binary classifications are performed, thus if dysplasia or ulceration was noted as benign by the pathologist, we classify this tissue as healthy epithelium. Thin (≤125 μm) and thick epithelium (>125 μm) regions (quantified by histology) were both grouped and treated as healthy epithelium as well in the binary classification analysis. The pixel-level annotations generated using this tool were stored for each sample as an image in the portable networks graphics format to avoid compression artifacts. These annotations were then coregistered with FLIm measurement (*x*, *y*) locations calculated by the aiming beam software.

FLIm measurements were coregistered to tissue annotations and analyzed only at regions directly guided by histopathology (annotated pixels) or within a 10-pixel distance from an annotation. Ten pixels correspond to less than 0.06% of the FOV (field of view) width captured using the system’s camera. This approach was used consistently across all patients with no patient-level fine-tuning applied. Measurements near heterogeneous tissue conditions (i.e. boundaries between cancer and healthy tissue) were excluded by removing any measurement within a 10-pixel radius of multiple disparate tissue conditions. A larger exclusion radius would remove a large quantity of data points from the analysis stage of this initial study. This filtering process was applied consistently across all patients. Performing both of these steps helps to mitigate tissue labeling errors.

A SNR threshold of 30 dB was applied across all spectral channels. Outlier removal was performed prior to any univariate statistical analysis for each FLIm parameter using a median absolute deviation (MAD) filtering approach [32], where parameter values ±2.5 MAD from their respective median were removed for each patient. This MAD filtering procedure was performed separately for *in vivo* pre-resection scans, *ex vivo* scans, and cavity scans. Spectral channel 4 was excluded from analysis due to the poor signal properties observed, thus six FLIm parameters were evaluated in this study: average fluorescence lifetime and spectral intensity ratio from spectral channels 1–3. Outlier removal and SNR filtering removed 0.5% and 2.5% of data points respectively.

#### 2.3.4 | Statistics and discrimination metrics

Statistical tests and discrimination metrics were implemented to quantify FLIm’s ability to distinguish between tissue conditions (e.g. healthy epithelium and cancer). In each case we compare measurements for healthy tissue and cancer unless otherwise stated. Statistically, we make no assumptions of a normal distribution for acquired FLIm data, however, we assume independence and equal variance, therefore we sought to evaluate our results with the Wilcoxon rank sum test, a non-parametric statistical method for significance testing [33, 34]. Receiver operating characteristic (ROC) analysis and precision-recall curve analysis were performed for each FLIm parameter to calculate a set of discrimination metrics.

Once calculated for a given variable, area-under-the-curve (AUC) and average precision (AP) provide a comprehensive overview of its discriminative power. Average precision is influenced more by the performance of the positive class (eg, cancer) and can highlight poor discrimination even if the dataset is imbalanced between classes (i.e. majority healthy), while AUC treats each class with equal importance. For each patient, analysis was performed separately for each specimen context (*in vivo* prior to tissue resection, *ex vivo* after tissue resection for the excised specimen, and *in vivo* for the cavity where the tumor was removed).

#### 2.3.5 | Multiparameter discrimination

Linear discriminant analysis (LDA) was performed to investigate if a weighted linear combination of the six calculated FLIm parameters can provide better discrimination of tissue types than individual FLIm parameters. This analysis was performed separately for each patient and tissue context as the focus of this work was on intrapatient contrast sources in FLIm rather than the development of a generalized classifier. The LDA variable was calculated for each case through singular value decomposition, minimizing the intraclass variance and maximizing the interclass variance. The optimized set of weights was applied to the FLIm data for a given scan before min-max scaling was performed, producing a set of LDA variables in the range of 0.0–1.0 for this scan, which are used to distinguish healthy tissue from cancer. AUC and AP were calculated for this LDA variable, allowing for a direct comparison with each individual FLIm parameter in terms of discriminative power. The objective here was not to train a generalized classifier but to compare single parameter and multiparameter tissue discrimination approaches within individual patients.

## 3 | RESULTS

FLIm measurements acquired from patients (n = 10) and subsequently analyzed generated a total of 42 777 FLIm data points coregistered with histopathology. 13 765 of these data points were associated to cancer and the remaining 29 012 to healthy tissue (epithelium and tumor bed). This dataset includes nine *in vivo* pre-resection scans, nine *ex vivo* post-resection scans, and seven post-resection tumor bed scans, for which no residual cancer was observed. Not all scan types were acquired for all patients due to changes in surgical plans during the OR acquisition process. Patient 9 did not have cancer, thus the patient was omitted from Figures 5 and 6 accordingly as intrapatient tissue type discrimination cannot be performed; a tumor bed scan however was successfully acquired and is included in the analysis. Table 1 summarizes the patient information involved in this study.

Here, detailed results (*in vivo* and *ex vivo* epithelial scans, as well as tumor bed scans) from two case studies (“A” and “B”), a summary of the statistical significance and discriminative metrics for the entire 10-patient cohort, and a comparison of normal tissue FLIm signatures between the three imaging contexts (pre-resection *in vivo*, post-resection *ex vivo* specimen, and post-resection cavity *in vivo*) are presented. These case studies were selected for the following reasons: (a) they highlight different levels of single parameter contrast (both *in vivo* and *ex vivo*) and show how limited contrast can be overcome through a multiparameter LDA approach when necessary and (b) distinct tissue conditions (ie, levels of heterogeneity) are observed in the histopathology for each case.

Each case study presents heat map visualizations of all six FLIm parameters with the associated AUC and AP score, violin plots (a non-parametric data visualization method which includes both a box plot and a kernel density plot) for all six FLIm parameters with statistically significant change (*P* < .001) highlighted, a heat map visualization of the multiparameter LDA variable along with AUC and AP score, and the associated ground truth drawn from histopathology coregistration (both high-level and fine-grained labels). Following the *in vivo* and *ex vivo* scan for each case, the FLIm profile of the imaged tumor bed scan is presented. Violin plots (a nonparametric method) were employed as a normal distribution cannot be assumed for FLIm data, while the kernel density plot included can highlight a multimodal data distribution. For *in vivo* pre-resection and *ex vivo* scans, the aim was to evaluate whether neoplastic changes in epithelial tissue diagnosed by conventional histopathology can be differentiated from surrounding healthy epithelial tissue, while for the post-resection cavity scan, the goal was to evaluate changes in optical parameters due to cauterization/surgical injury.

### 3.1 | Case study A

**FIGURE 3:**
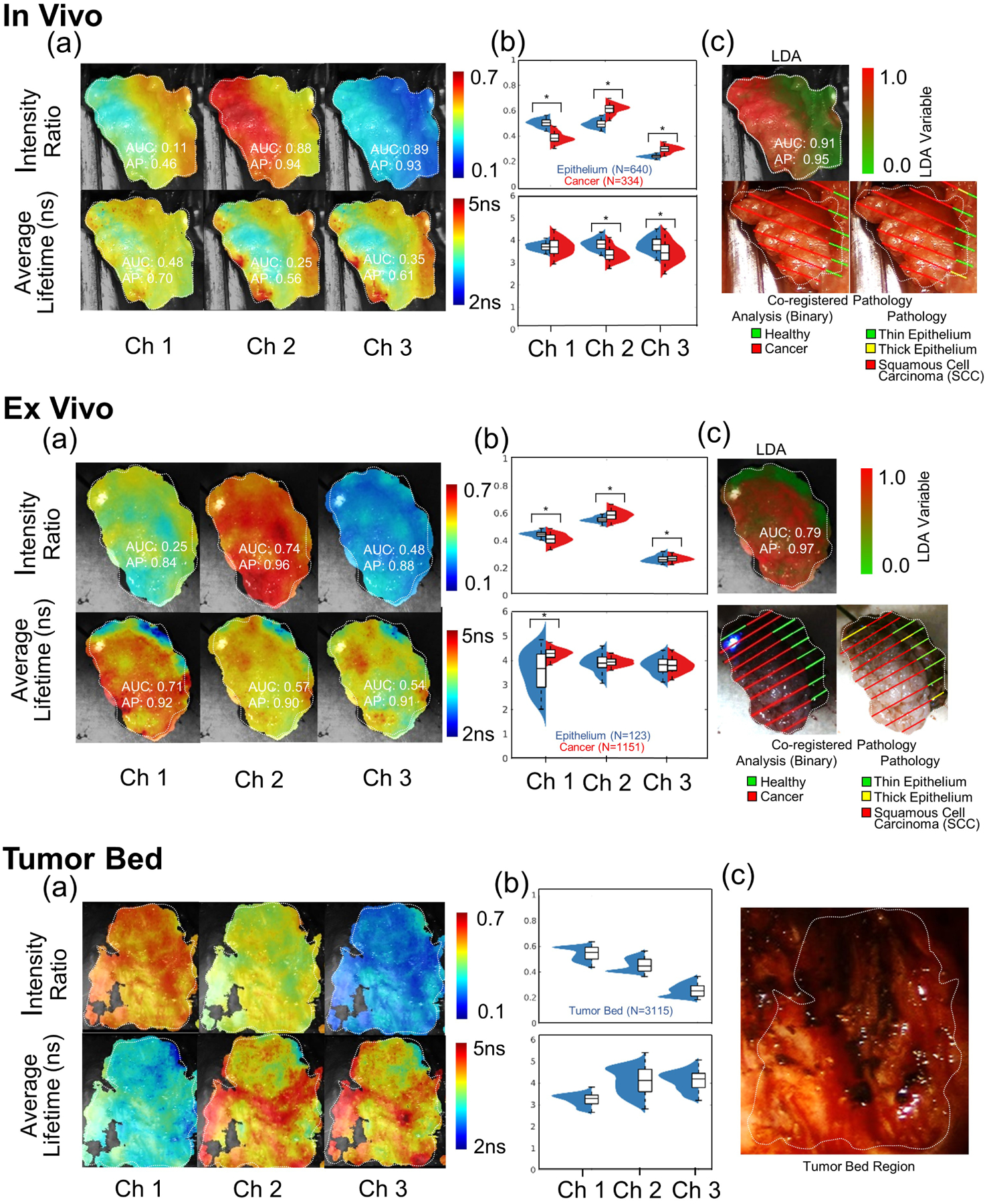
Case study A (Patient 8): Upper panel: pre-resection *in vivo* scans. (A) Heat map visualizations of six fluorescence lifetime imaging (FLIm) parameters (three intensity ratios and three average lifetime values with corresponding area-under-the-curve [AUC] and average precision [AP] values). (B) Violin plots for the six FLIm parameters (statistical significance marked (*) for (*P* < .001). (C) Heat map visualization of the linear discriminant analysis (LDA) variable (corresponding AUC and AP values) with the coregistered pathology results (endothelial surface) mapped on the da Vinci camera while light video image. Thin (≤125 μm) and thick epithelium (>125 μm) regions (quantified by histology) were analyzed concurrently for the LDA analysis. Middle panel: post-resection *ex vivo* scans. (A) Heat map visualizations of six FLIm parameters (three intensity ratios and three average lifetime values with corresponding AUC and AP values). (B) Violin plots for the six FLIm parameters (statistical significance marked [*] for *P* < .001). (C) Heat map visualization of the LDA variable (corresponding AUC and AP values) with the coregistered pathology results mapped on the *ex vivo* mounted camera white light video image. For LDA analysis, thin and thick epithelium regions were analyzed concurrently. Bottom Panel: post-resection *in vivo* tumor bed scans. (A) Heat map visualization for six FLIm parameters. (B) Violin plots for six FLIm parameters depicting the broad distribution of cauterized healthy submucosa. *Note*: No residual tumor (positive margins) were found corresponding to the tissue deep margin surface. (C) White light image of the tumor resection bed

Case study A (Figure 3) presents FLIm measurements for Patient 8. Statistically significant change (*P* < .001) was observed between tissue conditions for five FLIm parameters in the *in vivo* pre-resection scan and four FLIm parameters in the *ex vivo* post-resection scan. LDA improved both AUC and AP score for both scans compared to the best performing individual parameter in each case. For the *in vivo* scan, the use of the LDA variable improved AUC and AP by just 0.02 and 0.01 respectively, suggesting that when good single-parameter contrast is observed (ie, AUC of 0.89) that a multiparameter approach (LDA) only results in marginal improvement. For the *ex vivo* scan, the use of the LDA variable improved AUC and AP by 0.04 and 0.01 respectively, these marginal improvements due to the already strong single parameter contrast observed (AUC of 0.74). A bimodal distribution was observed within the tumor bed for intensity ratio in CH1, CH2, and CH3, suggesting that distinct tissue conditions are present.

### 3.2 | Case study B

**FIGURE 4:**
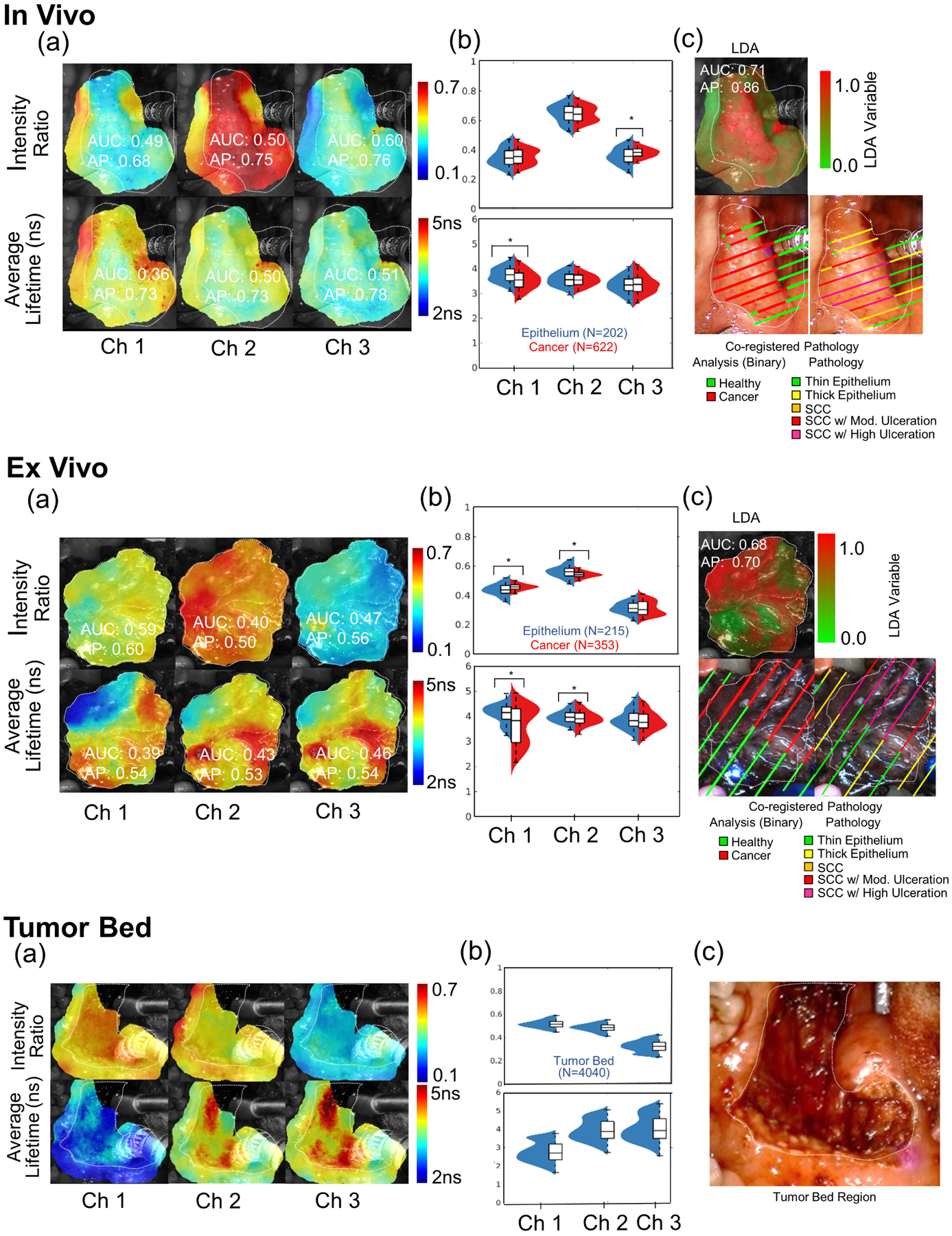
Case study B (Patient 5): Upper panel: pre-resection *in vivo* scans. (A) Heat map visualizations of six fluorescence lifetime imaging (FLIm) parameters (three intensity ratios and three average lifetime values with corresponding area-under-the-curve [AUC] and average precision [AP] values). (B) Violin plots for the six FLIm parameters (statistical significance marked [*] for *P* < .001). (C) Heat map visualization of the linear discriminant analysis (LDA) variable (corresponding AUC and AP values) with the coregistered pathology results (endothelial surface) mapped on the da Vinci camera white light video image. Thin (≤125 μm) and thick epithelium (>125 μm) regions (quantified by histology) were analyzed together for the LDA analysis. Middle panels: post-resection *ex vivo* specimen scans. (A) Heat map visualizations of six FLIm parameters (three intensity ratios and three average lifetime values with corresponding AUC and AP values). (B) Violin plots for the six FLIm parameters (statistical significance marked [*] for *P* < .001). (C) Heat map visualization of the LDA variable (corresponding AUC and AP values) with the coregistered pathology results mapped on the *ex vivo* mounted camera white light video image. For LDA analysis, thin and thick epithelium regions were analyzed together under epithelium, and SCC with and without ulcerations were analyzed together under the cancer group. Bottom panels: post-resection *in vivo* tumor bed scans. (A) Heat map visualizations for six FLIm parameters. (B) Violin plots for six FLIm parameters depicting the broad distribution of cauterized healthy submucosa. *Note*: No residual tumor (positive margins) were found corresponding tissue specimen (deep margins surface). (C) White light image of the tumor bed region. SCC, squamous cell carcinoma

Case study B (Figure 4) presents FLIm measurements for Patient 5. Statistically significant change (*P* < .001) is observed between tissue conditions for two FLIm parameters in the *in vivo* pre-resection scan and four FLIm parameters in the *ex vivo* post-resection scan. LDA improved both AUC and AP score in both scans compared to the best performing individual parameter in each case. For the *in vivo* scan, the use of the LDA variable improved AUC and AP by 0.11 and 0.12 respectively, highlighting the advantage of a multiparameter approach when single parameter contrast is not strong (ie, AUC of 0.60). For the *ex vivo* scan, the use of the LDA variable improved both the AUC and AP by 0.11, once again highlighting the benefit of a multiparameter approach. A bimodal distribution is observed within the tumor bed for average lifetime in channels 1–3 and intensity ratio CH1, suggesting heterogeneity within this region.

### 3.3 | Patient-level significance, ROC-AUC and average precision comparisons

Statistically significant change (*P* < .001) between healthy epithelium and cancer is observed for at least one FLIm parameter in all *in vivo* pre-resection scans and 8/9 *ex vivo* post-resection scans.

**FIGURE 5:**
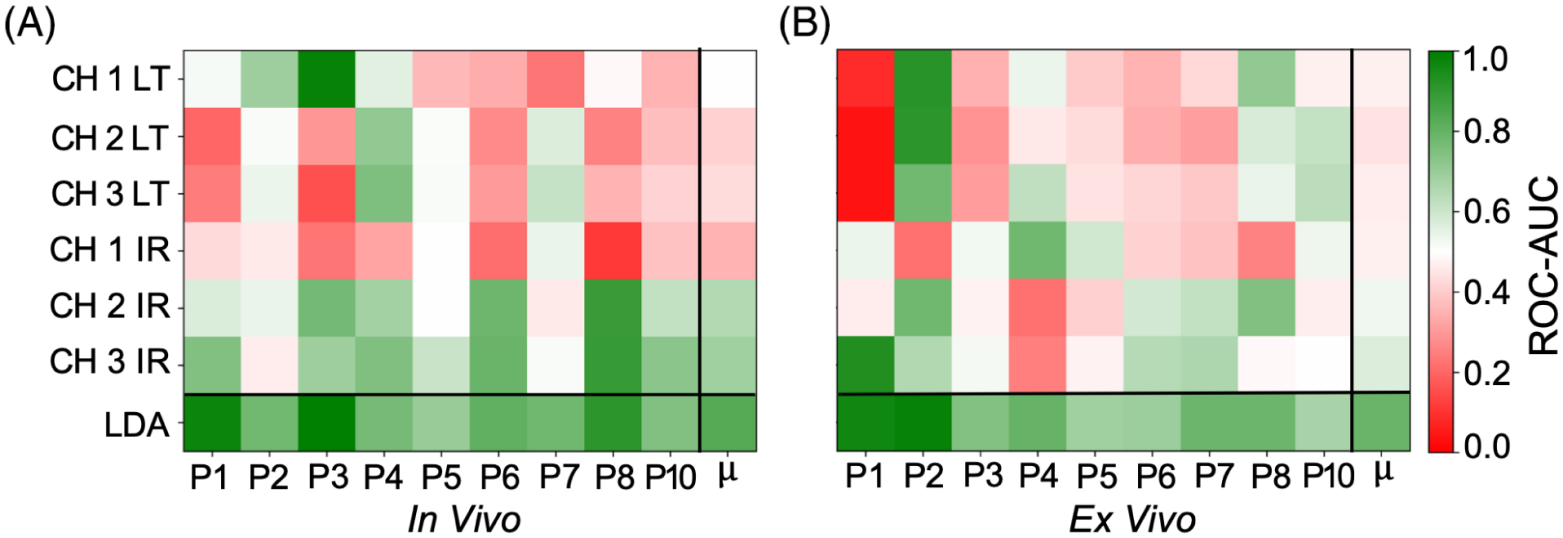
A comparison of patient-level ROC-area-under-the-curve [AUC] performance for (A) *in vivo* pre-resection scans and (B) *ex vivo* post-resection scans. μ corresponds to the mean performance for each parameter including the linear discriminant analysis (LDA) variable. For each scan type, use of the LDA variable resulted in superior AUC to the best performing signal parameter, with a 0.07 ± 0.03 mean increase observed for the *in vivo* pre-resection scans and a 0.06 ± 0.03 mean increase observed for the *ex vivo* scans. A single parameter AUC score greater than 0.70 was observed for 6/9 *in vivo* pre-resection scan, and 4/9 *ex vivo* scans

A comparison of patient-level ROC-AUC scores for the *in vivo* pre-resection scans and *ex vivo* scans is presented in Figure 5. The main sources of contrast among the six FLIm parameters varied among patients for each scan context. In all scans, the use of the LDA variable resulted in superior AUC score, with a 0.07 ± 0.03 mean increase observed for the *in vivo* pre-resection scans and a 0.06 ± 0.03 mean increase observed for the *ex vivo* post-resection scans. In terms of single parameter discriminative performance, the highest single parameter mean AUC was observed for CH3 intensity ratio in both scan contexts. A single parameter AUC score greater than 0.70 was observed for 6/9 *in vivo* pre-resection scan and 4/9 *ex vivo* post-resection scans.

**FIGURE 6:**
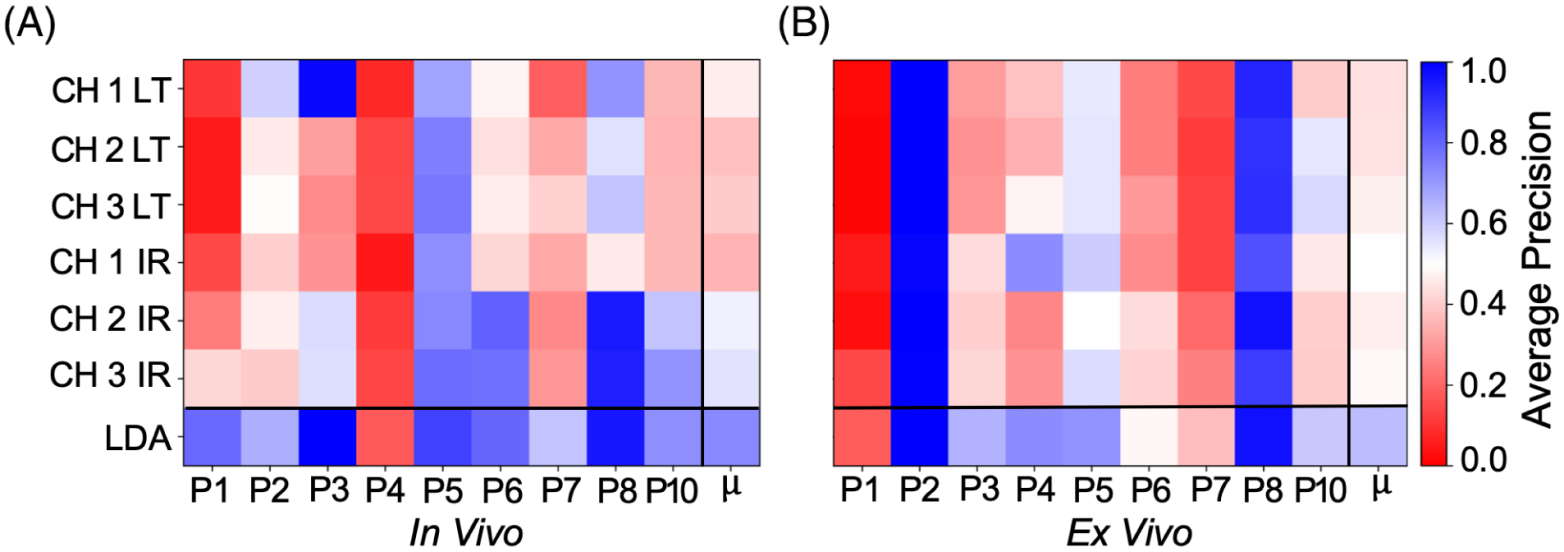
A comparison of patient-level average precision performance for (A) *in vivo* pre-resection scans and (B) *ex vivo* post resection scans. μ corresponds to the mean performance for each parameter including the linear discriminant analysis (LDA) variable. For each scan type, the use of the LDA variable resulted in superior AP to the best performing signal parameter, with a 0.08 ± 0.06 mean increase observed for the *in vivo* pre-resection scans and a 0.06 ± 0.03 mean increase observed for the *ex vivo* scans. A single parameter AP score greater than 0.70 was observed in 6/9 *in vivo* pre-resection scans and 3/9 *ex vivo* scans

A comparison of patient-level average precision for the *in vivo* and *ex vivo* post-resection scans is presented in Figure 6. As observed for ROC-AUC analysis, the main sources of contrast varies between patients for both scan contexts. The use of LDA resulted in a superior overall AP score, with a 0.08 ± 0.06 mean increase observed for the *in vivo* pre-resection scans and a 0.06 ± 0.03 mean increase observed for the *ex vivo* post-resection scans. In terms of single parameter discriminative performance, the highest single parameter mean AP was observed for CH3 intensity ratio in the *in vivo* pre-resection scans and CH1 intensity ratio for the *ex vivo* scans. A single parameter AP score greater than 0.70 was observed in 6/9 *in vivo* pre-resection scans and 3/9 *ex vivo* post-resection scans.

### 3.4 | Comparison of healthy epithelium in the tonsil region

**FIGURE 7:**
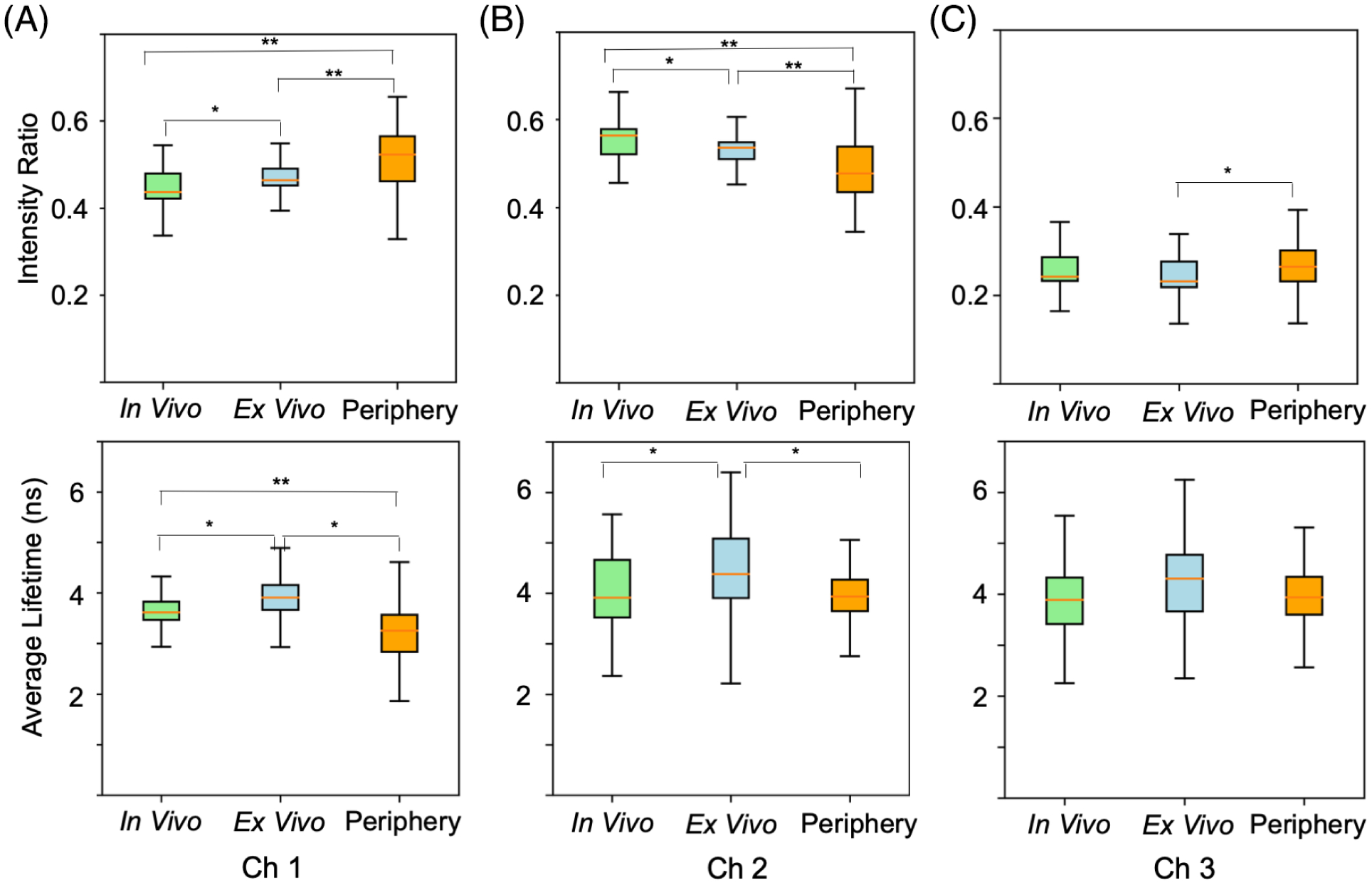
Comparison of fluorescence lifetime imaging parameter values for all measurements of non-cauterized healthy epithelial tissue (tonsil regions, n = 9 patients) taken *in vivo* pre-resection (n = 5606 measurements), *ex vivo* post-resection (n = 7252 measurements), and *in vivo* post-resection (peripheral to tumor bed) (n = 4060 measurements). Intensity ratios and average lifetimes for (A) CH1 fluorescence emission, (B) CH2 fluorescence emission, and (C) CH3 fluorescence emission. Cohen’s *d* [36] effect size (ES) is computed between imaging contexts. *ES > 0.5; **ES > 0.8

Figure 7 illustrates the range of FLIm parameter values for all measurements of noncauterized healthy epithelium taken in three distinct experimental conditions (contexts): *in vivo* pre-resection (n = 5606 measurements), *ex vivo* post-resection (n = 7252 measurements), and *in vivo* post-resection (peripheral to tumor bed) (n = 4060 measurements). Box plots (a non-parametric method) were used to display the values distribution as a normal distribution for FLIm data cannot be assumed. All measurements were performed for the tonsil region of the oral cavity (n = 9). Data from any non-tonsil patient (i.e. Patient 9) is excluded from this analysis to restrict the focus to a single anatomical location (tissue type). The same preprocessing steps are performed as for previous experiments.

Due to the high number of measurements included in this analysis (n > 10 000), *P* values are not computed as these statistics are shown to always indicate significance as N grows very large [35]. Alternatively, Cohen’s *d* [36] effect size (ES) was computed to overcome this high sample size and quantify parameter change in healthy epithelium between imaging contexts. Higher ES values are observed between healthy epithelium contexts for intensity ratio parameters compared to average lifetimes. Channel 3 has the smallest set of ES values between healthy epithelium contexts for both average lifetime and intensity ratio, indicating this channel’s FLIm profile may be the most robust to these context changes. For average lifetime in channels 2 and 3, there is a small ES observed between pre-resection *in vivo* and post-resection peripheral tissue (*in vivo*), suggesting a consistent FLIm profile for these parameters before and after resection. For channel 1 average lifetime, a high ES is observed between healthy epithelium in all three imaging contexts, suggesting this parameter is less robust to these changes with respect to a healthy epithelium imaging context.

### 3.5 | Comparison of tumor bed with pre-resection healthy epithelium and cancer

Average lifetime parameters observed in the tumor bed (*in vivo*) (n = 19 567) are compared with those observed pre-resection for both *in vivo* healthy epithelium (n = 6151) and *in vivo* cancer (n = 3760) across the entire 10-patient cohort. Cohen’s *d* [36] ES is once again employed due to the high sample size. For channel 1 average lifetime an ES of 1.45 and 1.5 are respectively observed when comparing tumor bed with healthy epithelium and cancer, suggesting a consistent change for this parameter when compared to both tissues. Conversely, for average lifetime in channels 2 and 3, no ES value greater than 0.36 is observed when comparing tumor bed with healthy epithelium and cancer, suggesting a less prominent change for these channels.

## 4 | DISCUSSION

Conventional oral and oropharyngeal surgery relies on visual inspection of changes of the epithelial surface and palpation to determine tumor margins prior to *en bloc* surgical resection. Intraoperative cancer assessments are primarily based on histopathologic analysis of the excised tissue specimens and/or biopsy samples. This process has inherent limitations: (a) it is subjective as it is based on the experience of the surgeon and pathology team, (b) the processing of frozen sections are not performed in real-time, thus introducing waiting time to the procedure, and (c) is subject to sampling error (which might not detect small infiltrative cancers in the initial sectioning for frozen section pathology assessments). Moreover, during TORS procedures, the surgeon lacks tactile cues. This current study demonstrates that FLIm, a label-free spectroscopic imaging technique, can be easily integrated with conventional TORS procedures and is inherently insensitive to illumination artifacts such as operating room light. Our results demonstrate that a set of FLIm-derived parameters can be used to distinguish between oropharyngeal cancer and healthy surrounding tissues both *in vivo* prior to surgical resection and *ex vivo* in excised *en block* tissue specimens. Our results establish the initial feasibility for FLIm’s potential to aid in real-time cancer delineation, tissue resection decision-making, and validation of the adequacy of resection.

First, we sought to investigate the use of FLIm to provide intrapatient contrast between healthy surface epithelium and cancer in two scenarios: (a) *in vivo* prior to resection and (b) *ex vivo* post-resection in surgically excised specimens (Figures 3–6). The former is to evaluate FLIm’s potential for real-time guidance of TORS procedures (decision-making) while the latter is to evaluate FLIm’s potential for use as an intraoperative pathology tool. While the number of individual parameters able to resolve healthy tissue from tumor varies, for 9/9 *in vivo* pre-resection scans and 8/9 *ex vivo* scans, we show that at least one FLIm parameter shows statistically significant difference (*P* < .001) per patient between healthy epithelial tissue and cancer. The experimental context by which data was acquired (*in vivo* or *ex vivo*) and tissue heterogeneity (e.g. variable thickness of the epithelial layer, ulcerations within tumor mass) appears to play a role in the number of parameters needed for discrimination. Overall, more parameters are needed for discrimination when more complex histopathological features were observed for a given patient. We highlight that a weighted linear combination of all six FLIm parameters by LDA provides superior discrimination of tissue conditions in both scenarios. Intensity ratio parameters are shown to have superior discriminative power to average lifetimes within this study in terms of mean AUC and AP scores in both scenarios, but average lifetime parameters do contribute to the performance of the LDA. A better FLIm-based diagnostic assessment can be achieved *in vivo* vs. *ex vivo* as demonstrated by the AUC and AP values. However, it is to be noted that the scaled LDA parameter is not a probability score for a given condition and should not be interpreted as such.

Second, we sought to evaluate the use of FLIm as a means to detect potential residual cancer in the deep margins at the locations where the tumor was surgically excised (tumor bed) and to investigate the fluorescence properties of healthy and/or electrocauterized submucosa (Figures 3 and 4, bottom panel). Out of the 10-patient cohort investigated, no patients presented with residual cancer following their procedure; as we continue enrolling patients in this research study, we anticipate that some patients will present with residual cancer and we subsequently wish to investigate FLIm’s ability to detect residual tumor on the electrocauterized submucosa during cavity scans. However, we were able to make interesting observations. FLIm parameters from tumor bed (healthy electrocauterized submucosa) measured *in vivo*, in particular average lifetime values in CH1, were significantly lower when compared to the values obtained for tumor measured *in vivo* pre-resection in the same patient. This trend was observed for all patients (CH1: ES of 1.45) indicating that CH1 (associated with the fluorescence emission of matrix proteins) might be able provide a means of contrast if residual tumor is present. For CH2 and CH3, an ES of 0.15 and 0.29 was observed, indicating little effect. We also noted that the average lifetime values of healthy electrocauterized submucosa were also significantly lower relative to healthy epithelium (CH1: ES of 1.5).

Third, we study the effect of the surgical procedure and potential hemostasis on healthy epithelium (Figure 7). Thus, we analyzed changes in FLIm parameters in three imaging contexts: (a) before the surgical procedure *in vivo*, (b) after *en bloc* tissue excision *ex vivo*, and (c) after the surgical procedure *in vivo* at the margins (peripheral to tumor bed). Current results indicate that FLIm parameters for healthy and pathological tissue changes with imaging context; this phenomena is expected due to the inherent sensitivity of endogenous fluorophores to their local microenvironment [37]. The literature demonstrates that biological tissue is under tremendous stress when it is surgically separated from the body [38], particularly due to the loss of blood supply and oxygenation changes following resection. Such conditions lead to a rapid shift (on the order of minutes) [39] of tissue metabolism towards anaerobic respiration [40], and rapid cell death [40], which will manifest with changes in tissue autofluorescence properties following excision. It has also been established that the molecular changes induced during and after tumor resection are heteromorphic (exhibiting unique differences among patients) [38, 39], while also demonstrating dependence on the anatomical tissue resected [38]. In addition to the aforementioned changes in surgically excised specimens, the healthy tissue surrounding the periphery of a surgical resection bed also changes as a result of the procedure, where surgery introduces a cascade of biological responses due to injury [41]. In particular, surgery creates a hypermetabolic response, induces catabolic metabolism changes, creates local vasodilation, and initiates other inflammation-mediated biochemical changes [41]. These findings help define the basis for FLIm’s parameters tissue context dependence, thus highlighting the motivation for analyzing each scan context separately. Additionally, the tumor bed is composed of submucosal tissue, which has different molecular and morphological properties compared to mucosa, and thus requires separate analysis.

This work demonstrates that intensity ratios are more likely to vary with imaging context. With respect to average lifetime, results show that CH1 (most sensitive to changes in matrix protein’s fluorescence), is most affected by the imaging context, whereas the lifetime parameters from CH2 and CH3 (associated with metabolic changes) are less affected. In particular, no major differences were observed for measurements performed *in vivo* pre- and post-resection. However, changes were observed for surgically excised specimens. These findings suggest a potential recovery of metabolic features of the epithelial tissue at the periphery of the tumor bed, but as expected, irreversible changes take place in the excised tissue specimens.

Although CH3 intensity ratio overall enabled the best separation of cancer vs. healthy tissue for this dataset, it is important to note that lifetimes were still very informative in distinguishing healthy tissue from cancer and in some cases, offered the best tumor vs. healthy contrast on a patient-by patient basis. For example, for patient 2, CH1 lifetime provided the strongest contrast between healthy tissue and cancer. When coupled with LDA, even if intensity ratio enabled the best data class separation, lifetimes bolstered the overall cancer vs. healthy tissue discrimination capacity for all patients.

In clinical practice, we envision that FLIm will leverage weighed combinations of all channels, using both intensity and lifetime data, to detect cancer. Having at least one channel which provides adequate tumor vs. healthy contrast is not required for cancer delineation; based on the results of this study, there is no single FLIm parameter that can distinguish healthy tissue from cancer in all contexts, therefore a multiparameter approach is required. For example, in Figure 5, *in vivo* and *ex vivo* for patient 7, there is no single metric from one channel that gives a significant difference, however after using weighted combinations of the FLIm metrics in an LDA, adequate tumor vs healthy contrast is achieved.

Among the data collected, differences are observed among which individual FLIm parameters provides the best healthy vs. cancer discrimination. These differences may be explained by the intrinsic differences between patients, as well as the aforementioned heteromorphic molecular changes for excised specimens. Because FLIm uses low penetration 355 nm UV light in our study, we detect surface-level features which are understood to vary between patients based on a number of factors including age, different anatomical locations, variables such as oral cavity health (impacted by tobacco and nicotine), variable thicknesses of the epithelium, the degree of malignancy of the tumor, and other biological factors. This highlights the importance of developing a large working database to assess the extent of interpatient variability.

This manuscript demonstrates that a set of FLIm parameters can always achieve contrast between healthy and tumor tissue for a 10 patient cohort. This is an important first step towards training a general classifier since discrimination is only possible if the FLIm method itself is capable of providing adequate healthy vs. tumor contrast. Having used this work as a baseline to demonstrate the feasibility of FLIm for intraoperative cancer detection, training a general classifier is the focus of our future work. In training a general classifier, our goal will be to incorporate a larger data population (after performing further clinical studies) which accounts for the diverse range of head and neck anatomic sites, cancer types, and inherent tissue differences across patients. Future work will also seek to evaluate how FLIm parameters are specifically affected by the presence of more granular features, such as the presence of ulceration, necrosis, low-grade and high-grade dysplasia.

Collectively, this preliminary study demonstrates that FLIm can be utilized as a label-free technique leveraging intrinsic contrast to enable discrimination between healthy tissue and cancer. We acknowledge that exogenous contrast agents may serve as a valuable complimentary technique to this method, however exogenous contrast agents have various limitations.

To cite two particular examples, indocynine green (ICG), an FDA approved fluorescent probe, is most commonly used in clinical practice, including robotic surgery. The integration of the “Firefly” module into the da Vinci Surgical System enables intraoperative visualization of blood flow and related perfusion employing ICG [42]. However, as ICG is not a molecularly targeted imaging probe [43], it relies on the presence of leaky capillaries [44], and lacks specificity [43] for early neoplastic lesions. Thus, this approach is not used for TORS. Recently, Panitumumab-IRDye800CW, a molecular targeted near-infrared fluorescence imaging agent, was reported as a means of aiding the surgeon in detecting intraoperatively close or positive margins in head and neck cancer patients [45]. Nevertheless, while very promising, this technique is based on a rather qualitative interpretation of fluorescence emission, relies on exogenous contrast, and requires a controlled light environment. To date, this agent has not been employed for TORS.

A variety of implementation challenges were encountered during this study. First, errors observed in the aiming beam position estimation (approximately 35% of measurements) required very time-consuming manual correction to be performed for all patients prior to data analysis to ensure correct coregistration, thus an improved method for point measurement localization will need to be developed in order to deploy this imaging modality for diagnostic use in a clinical setting. Second, the ground truth associated with each patient is limited in scope due to the histological coregistration process which restricts the amount of data points which can be used for analysis. Machine learning and transfer learning techniques will be investigated to overcome this data limitation, in addition to expanding the number of patients in our database. A third challenge arises from the three-dimensional (3D) nature of the scanned tissue region, particularly for *in vivo* scans, which makes it difficult to coregister the acquired measurement data using a 2D annotation and visualization pipeline. 3D visualization and coregistration techniques will be investigated in future work via augmented reality techniques.

Due to limitations imparted by the physics of autofluorescence, we remark that FLIm is only able to detect cancer present on exposed mucosal surfaces (within 200–300 μm depth from surface). Suitably, cancers originating at the depths of tonsillar crypts with a submucosal origin may evade detection; this fundamental limitation introduces the potential to miss the submucosal extent of known cancers and/or permit small submucosal tumors to evade detection when looking for unknown primary cancers. This penetration depth limitation can be addressed however through incorporation of the FLIm probe into a biopsy needle and/or coupling FLIm to a molecular-based exogenous probe.

Both *in vivo* and *ex vivo* FLIm can be informative in intraoperative settings. The *in vivo* pre-resection scan allows for FLIm to add additional information prior to initiating tumor resection to help account for the loss of tactile feedback and visual cues imparted by robotic surgery. This can enable better initial resection of tumor tissue and permit more conservative preliminary resection practices. The *ex vivo* scan however is also important as the excised specimen can be leveraged for a confirmatory verification that all tumor has been appropriately removed from the patient. Both *in vivo* and *ex vivo* scans can be implemented together as they are label free, can complement the surgical verification process, and are relatively quick to implement *in situ*. FLIm has potential in many other areas of clinical practice beyond otolaryngology applications, including neurosurgery [21] and breast cancer surgery [46] as previously reported.

## 5 | CONCLUSION

The results of this study suggest that fluorescence lifetime imaging (FLIm) has the potential to be developed into a diagnostic tool for clinical cancer applications of the oropharynx. Once such a system is implemented and extensively validated, this technique can help to circumvent the issues posed by the lack of tactile feedback associated with robotic surgical platforms and assist in cancer delineation. The real-time dynamics of FLIm signal acquisition and processing has potential to address the time-consuming nature of conventional intraoperative diagnostic standards (e.g. frozen section biopsies) while also remaining entirely non-invasive. However, we acknowledge that this technology is still early in development and requires further investigation. In order for FLIm to be used as a universal transoral diagnostic standard, the biological complexity of cancer and the fundamental biochemical variability across patients needs to be considered in order to develop a generalized combination of signal parameters which can be utilized for diagnostic decision-making. Machine learning methods will be investigated as means to produce such a generalized model and define the fluorescence decay signatures of specific conditions. We anticipate that continued rigorous research in this area will enable the generation of larger and more robust data sets to better elucidate the extent of interpatient variability, and identify common autofluorescent properties which can be leveraged for pathology contrast.

## Figures and Tables

**TABLE 1:** Overview of patients, afflicted anatomical tissues, resulting pathologies, and residual cancer status

Patient	Anatomical location	Pathology diagnosis	Residual cancer status
1	Left lingual tonsil	Squamous cell carcinoma	No residual cancer
2	Left palatine tonsil	Squamous cell carcinoma	No residual cancer
3	Left palatine tonsil	Squamous cell carcinoma	No residual cancer
4	Left palatine tonsil	Squamous cell carcinoma	No residual cancer
5	Left palatine tonsil	Squamous cell carcinoma	No residual cancer
6	Right palatine tonsil	Squamous cell carcinoma	No residual cancer
7	Left palatine tonsil	Basaloid squamous cell carcinoma	No residual cancer
8	Right palatine tonsil	Squamous cell carcinoma	No residual cancer
9	Base of tongue	No cancer	No residual cancer
10	Right palatine tonsil	Squamous cell carcinoma	No residual cancer
